# Molecular endoscopic imaging for the detection of Barrett’s metaplasia using biodegradable inorganic nanoparticles: An ex-vivo pilot study on human tissue

**DOI:** 10.1371/journal.pone.0239814

**Published:** 2020-10-01

**Authors:** Shakil Ahmed, Andreas Kreft, Ezharul Hoque Chowdhury, Sultana Mehbuba Hossain, Peter R. Galle, Helmut Neumann

**Affiliations:** 1 Inner Medicine, University Medical Centre, Mainz, Germany; 2 Institute of Pathology, University Medical Centre, Mainz, Germany; 3 Jeffrey Cheah School of Medicine and Health Sciences, Monash University Malaysia, Subang Jaya, Malaysia; University of California Berkeley, UNITED STATES

## Abstract

**Background and study aims:**

Despite major technical advancements, endoscopic surveillance for detecting premalignant lesions in Barrett’s esophagus is challenging because of their flat appearance with only subtle morphological changes. Molecular endoscopic imaging (MEI) using nanoparticles (NPs), coupled with fluorescently labeled antibody permits visualization of disease-specific molecular alterations. The aim of this *ex vivo* study was to assess the diagnostic applicability of MEI with NPs to detect Barrett’s metaplasia.

**Patients and methods:**

Seven patients undergoing endoscopic surveillance of known Barrett’s esophagus were recruited. Freshly resected biopsy specimens were incubated with NPs coupled with FITC labeled Muc-2 antibodies and examined with MEI. Fluorescence intensity from Barrett’s mucosa and control specimens were compared, followed by histological confirmation.

**Results:**

Fluorescence signals, indicating the presence of goblet cells, were noted for traditional MEI using Muc-2 antibodies in Barrett’s intestinal metaplasia. Significantly stronger fluorescence signals were achieved with NPs coupled with FITC-conjugated Muc-2 antibodies. The results of MEI with NPs for the prediction of Barrett’s metaplasia correlated with the final histopathological examination in all the cases.

**Conclusions:**

Highly-specific NPs detected Barrett’s metaplasia more efficiently than conventional MEI in this first feasibility study. MEI was as effective as standard histopathology for identifying Muc-2 containing goblet cells for diagnosis of Barrett’s metaplasia. **(DRKS-ID: DRKS00017747)**

## Introduction

Barrett’s esophagus (BE) is the precursor of esophageal adenocarcinoma (EAC) and considered the eighth most common cancer in the world [1]. Molecular endoscopic imaging (MEI) has the potential to visualize disease-specific molecular alterations in BE [2]. Imaging agents that are specific for overexpressed molecular targets can play a significant role in early-stage diagnosis and overall management of the disease.

Nanoparticles (NPs) are particularly useful for diagnostic and therapeutic applications because of their unique physicochemical properties and non-toxicity. The conjugation of antibodies with NPs combines the specific and selective antigen recognition ability of the antibodies with the properties of the NPs. Nanoparticles have increased surface area to volume ratio allowing for greater surface functionality. Therefore, in order to visualize multiple targets or signaling pathways several specific ligands can be coupled with a single particle. Moreover, the size of NPs ranges from 5–100 nm, allowing for a higher rate of distribution *in vivo* as they can overcome biological barriers. The aim of this study was to assess for the first time the diagnostic applicability of MEI using antibodies and NPs in Barrett’s metaplasia.

## Patients/Materials and methods

### Preparation of fluorescently labeled antibody-conjugated nanoparticles

**Fig 1:**
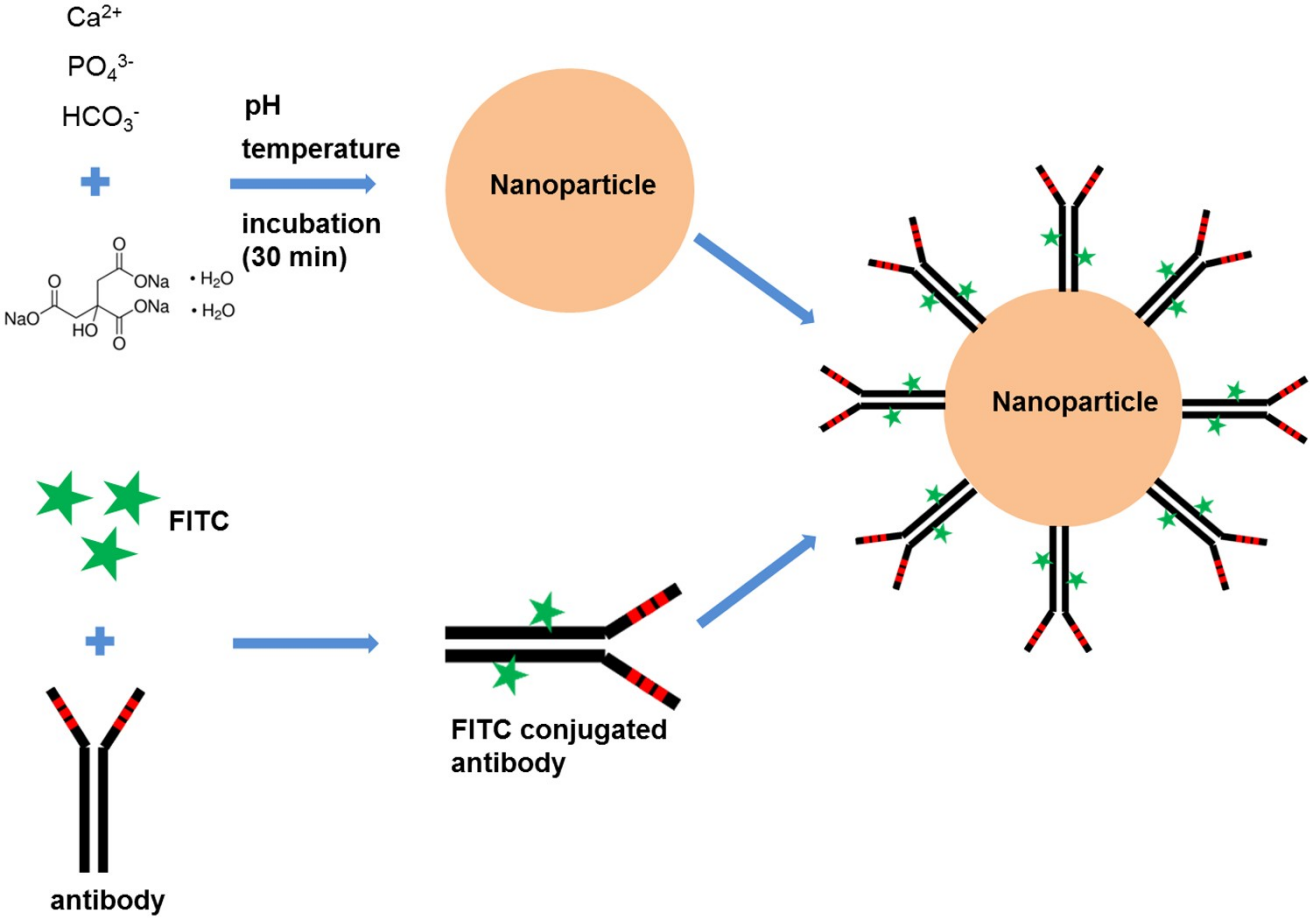
Preparation of antibody conjugated nanoparticles.

First, mouse monoclonal Muc-2 antibody (Abcam, Cambridge, UK) was covalently conjugated with fluorescein isothiocyanate (FITC) according to the manufacturer’s guidelines. Next, apatite-based pH sensitive inorganic CMCA (citrate-modified carbonate apatite) NPs were synthesized through the precipitation method [3] by adding 1 mM sodium citrate tribasic dehydrate and 4 mM exogenous Ca^2+^ to 1 mL fresh DMEM medium, followed by incubation at 37°C for 30 min. Turbidity was measured at wavelength 320 nm by UV-VIS spectrophotometer (Jasco, Oklahoma City, OK, USA) against fresh medium as a blank. Different amounts of FITC labeled antibodies were added to the NPs, followed by incubation at room temperature for 30 min (Fig 1), and then, maintained on ice.

**Fig 2:**
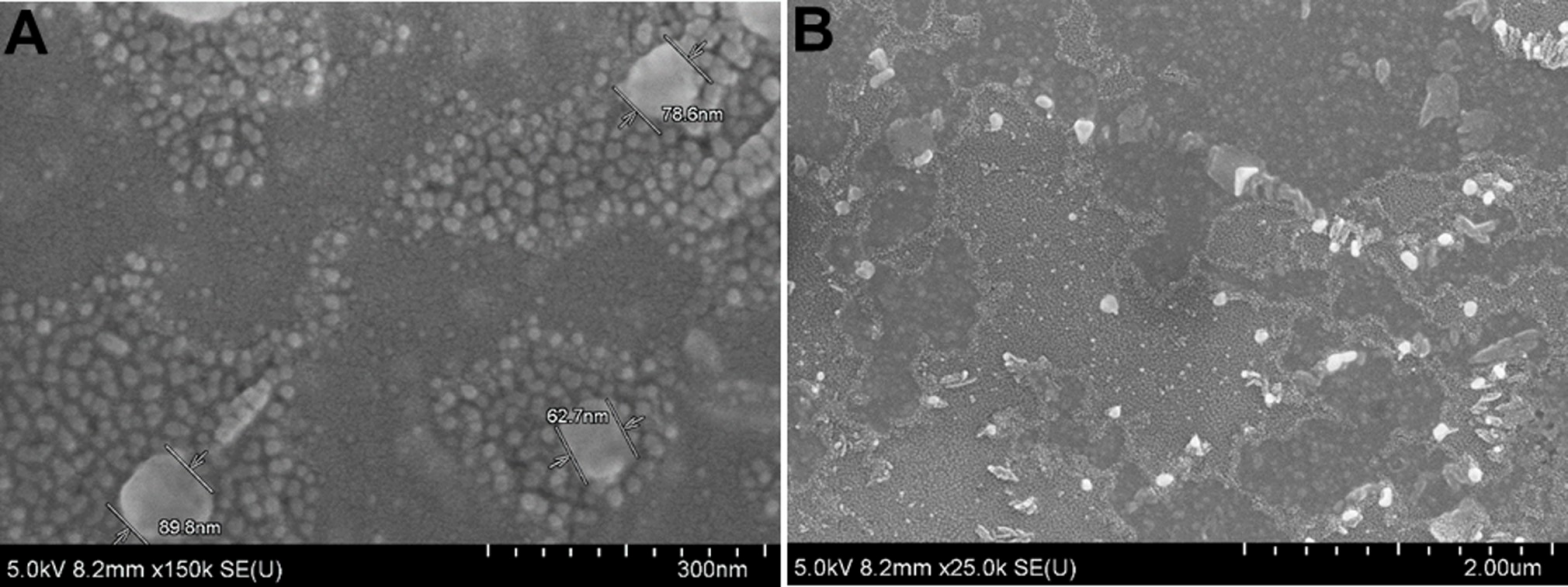
FE-SEM micrographs of nanoparticles at 300 nm (A) and 2 μm (B) scale.

The size, architecture, and chemical characteristics of NPs were studied using field emission scanning electron microscopy (Fig 2). The NPs were spherical in shape, and the diameter ranged from 10 to 90 nm, thus offering a large surface area for proper functionalization and fluorescent labeling.

### Endoscopic examinations

The pilot study was conducted on 7 human subjects. In the period between August 2018 and March 2019, patients with Barrett’s esophagus, according to the Montreal classification [4], were considered for study inclusion. Informed consent in written form was obtained from each patient prior to endoscopy. The study protocol was approved by the Ethics committee of Rhineland-Palatinate and has been registered for the study. Endoscopy procedures were performed under conscious sedation (e.g., propofol) with constant monitoring of vital signs. First, a careful inspection of the Barrett’s segment with high-definition white-light imaging (Fujifilm, Tokyo, Japan) was performed, followed by a careful inspection with virtual chromoendoscopy (Blue Light Imaging, Fujifilm, Tokyo, Japan) and optical magnification, if necessary. Following the endoscopic inspection, biopsies were obtained from Barrett’s segment and also from healthy tissues (control biopsies) and stored in PBS for subsequent MEI diagnosis. The biopsies were sent for the histopathological workup in 4% buffered formalin, thereby allowing diagnosis and comparison of exactly the same tissue. The histopathological workup was performed by a specialized gastrointestinal pathologist.

### *Ex-vivo* tissue examination through molecular imaging

The biopsy specimens were rinsed with ice cold PBS, incubated with FITC-labeled Muc-2 antibody (1–10 μg /mL) or NPs coupled with the same antibody for 2 min, washed again with PBS to remove the unbound antibody, and finally, imaged with a probe based confocal laser endomicroscope (Mauna Kea Technologies, France). The incorporated blue laser light had an excitation maximum of 488 nm and a detection maximum of 520 nm. The device has a field of view 240 X 200 μm, and lateral resolution of 1 μm. Images were collected at a scan rate of 12 frames / second and scanning field 30,000 pixels. The combined procedure, involving biopsy collection, staining, and imaging was performed within 30 min to ensure the preservation of the specimens for subsequent histopathology.

### Pathological examination

After *ex vivo* imaging, all biopsies were immediately fixed in 4% paraformaldehyde, and then, paraffin embedded, sectioned (~4-μm), and stained with hematoxylin and eosin (H&E) and periodic acid-Schiff stain (PAS). For the immunohistochemical (IHC) analysis of Mucin-2 expression, sections were deparaffinized, rehydrated, and heat-treated (100°C) in Dako EnVision^™^ FLEX Target Retrieval Solution, pH 9.0 (Dako, Glostrup, Denmark) for 25 min using Microwave. The slides were then immersed in 3% hydrogen peroxide solution for 10 min, blocked with 1% BSA for 30 min, and then, incubated overnight with the specific antibody (Muc-2, clone CCP58, Dako, ready to use) at 4°C. The slides were subsequently incubated with HRP-conjugated secondary antibody (Dako REAL^™^ EnVision^™^ Detection System, Rabbit/Mouse) for 30 min at room temperature; antibody binding was visualized with diaminobenzidine (Dako), and the slides were counterstained with hematoxylin. A section was stained with the same protocol, in which the primary antibody was replaced with the diluents, and it was considered as the control.

## Results

### Pilot *ex-vivo* study in BE patients

**Fig 3:**
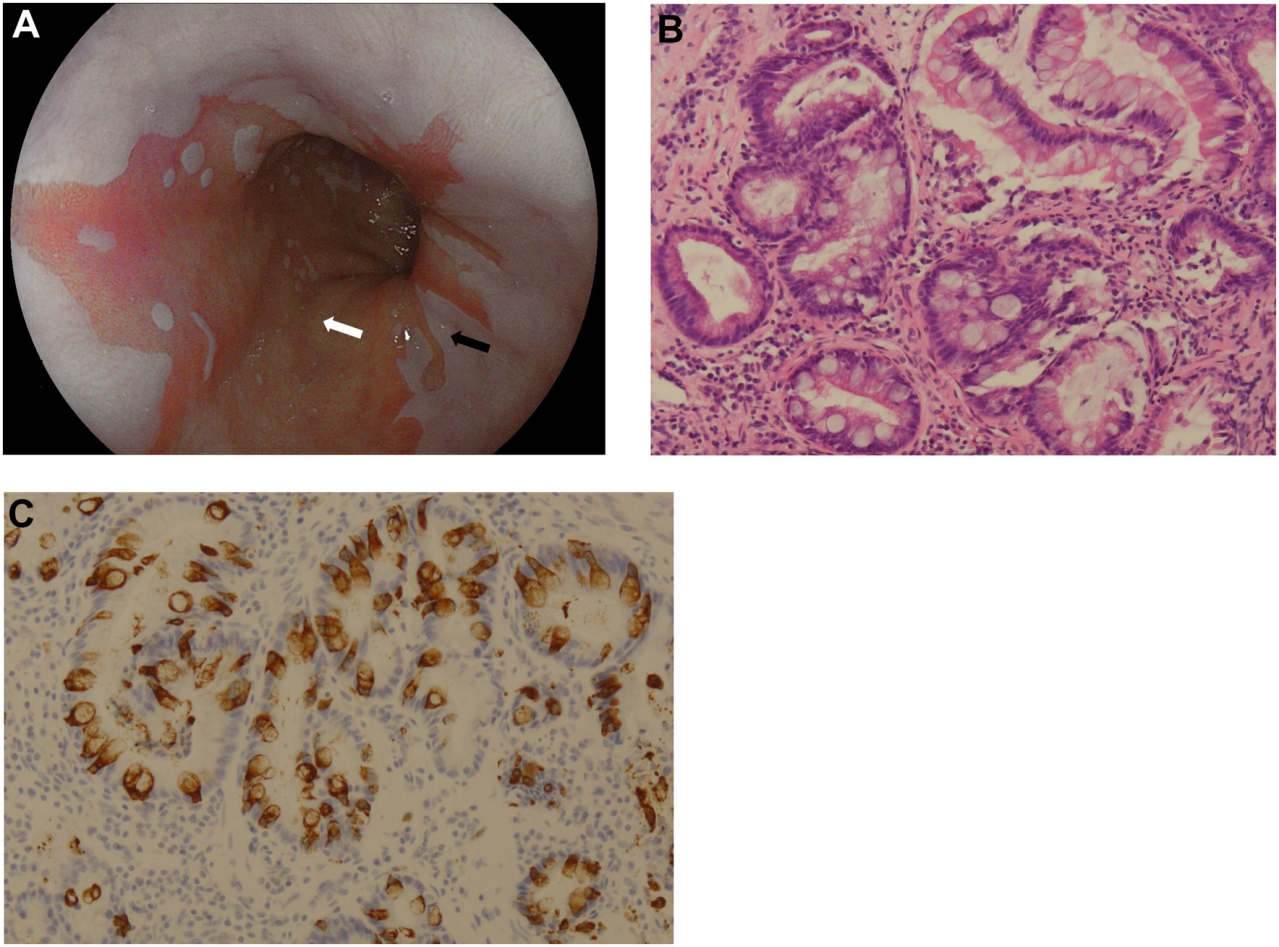
Endoscopic examination revealed columnar-lined epithelium (salmon red areas) in the inner layer of the esophagus in comparison with its neighboring white region (A). Histology was performed for all lesions. Biopsies taken from endoscopically detected lesions and squamous epithelium were analyzed through H&E staining and IHC. Microscopic images demonstrated the characteristic histological features of Barrett’s mucosa displaying numerous intestinal type goblet cells revealing apical Mucin dispersed among columnar cells (B) and were highlighted by Muc-2 IHC (C). No cytological atypia was found.

Gastroscopy was performed in all BE patients (Fig 3). A total of 26 biopsy specimens from 12 lesions were obtained from all patients. Table 1 shows the histological characteristics of the collected lesions.

**Table 1:** Characteristics of collected biopsies through histology.

Patient	Age of patient	Prag Classification	Lesion type	Definitive histology
1	64	C7M7	nodular	Barrett without dysplasia
2	64	C1M3	flat	Barrett without dysplasia
3	47	C0M1	flat	No Barrett metaplasia
4	63	C0M1	flat	No Barrett metaplasia
5	68	C1M1	nodular	No Barrett metaplasia
6	66	C3M5	nodular	Barrett without dysplasia
7	77	C0M1	flat	No Barrett metaplasia

#### Imaging of Mucin-2 in biopsy specimens using CLE

**Fig 4:**
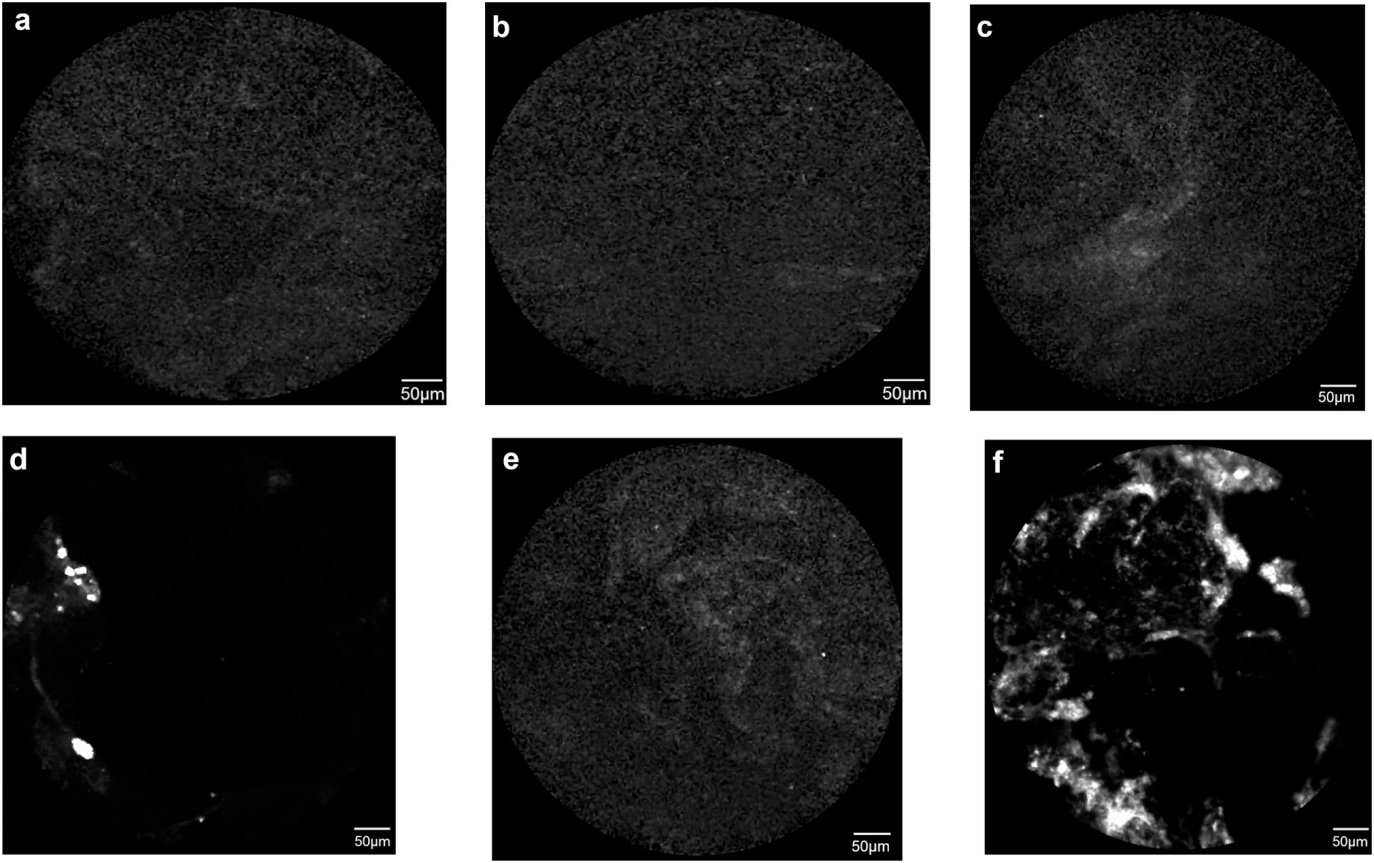
CLE imaging before antibody/NPs application to squamous epithelium (a) and Barrett’s esophagus (b) with no fluorescent signal. Similar results were achieved when squamous epithelium was treated with Anti Muc 2-FITC (c) and NP-Anti Muc 2-FITC (e). When Specimens from Barrett’s metaplasia were examined after incubating with FITC-labeled Muc-2 antibody (d) or NPs coupled with the same antibody (f), the fluorescence signal was detected in both the cases confirming the presence of Mucin 2 protein secreted by intestinal type goblet cells. A very strong fluorescence signal was noticed in a large area in the NPs treated specimens (f) because of signal amplification by NPs.

Confocal laser endomicroscopy (CLE) was carried out immediately after taking biopsies and incubation of the specimens with Ab-FITC or NP-Ab-FITC and compared with untreated specimens. The concentration of the molecular probe and total exposure time of tissue were kept constant to compare the fluorescence images qualitatively. No fluorescence signal was noted in the esophageal squamous epithelium specimens. In the case of Barrett’s mucosa, fluorescence signals were observed for all specimens undergoing either traditional MEI (Ab-FITC) or MEI using NPs (NP-Ab-FITC). Interestingly, significantly stronger fluorescence signals were achieved with NPs coupled with FITC-conjugated antibody (Fig 4). In the samples from the healthy regions, no fluorescence signals were observed (results not shown). For the diagnosis of Barrett’s metaplasia, MEI results with fluorescence signals were comparable with histopathological examination.

## Discussion

Here we described for the first time the diagnostic applicability of NPs for molecular endoscopic imaging (MEI) to detect BE. MEI targeting a goblet-cell specific Mucin has been shown to be effective for diagnosis of BE and demonstrated comparable results to standard immunohistochemistry.

Several studies have been carried out to detect gastrointestinal lesions using targeted imaging using fluorescent molecular probes [5–8]. However, the applicability of NPs in the detection of molecular lesions in gastrointestinal diseases remained untouched. The method is flexible and can also be applied to the prognosis of other diseases, simply by using disease-specific marker antibodies. The major advantage of using CMCA NPs is the ability to bind several antibodies on the surface to enhance the signals. This amplification could offset the insufficiency of specific markers in premalignant lesions. Moreover, they are inorganic; therefore, easy to prepare, narrow size distribution, and highly stable [9]. Since the constituents of NPs, carbonate apatite and citrate, form our body components, NPs can be considered clinically safe.

However, during *in vivo* study, NPs can be recognized by phagocytic cells and can be removed from the circulation. This can be overcome by sophisticated surface modifications, like PEGylation [10] or dextran coating [11]. Currently, it has been accepted that 10–100 nm is the optimal size for drug delivery systems. These NP formulations take advantage of the enhanced permeability and retention (EPR) effect in tumors and avoid elimination in the spleen [12]. As shown in Fig 2, the CMCA NPs also have a similar size range and hold great potential for the implementation of image guided therapy.

After endoscopy, MEI was performed with biopsies. No fluorescence signal was observed in squamous epithelium and in Barrett’s mucosa before antibody/NP-antibody treatment (Fig 4), indicating the specificity of the approach. Barrett’s specimens from the same site were examined in bed side after applying: i) FITC-labeled Muc-2 antibody, or ii) NPs coupled with the same antibody. The fluorescence signal was detected in both the cases. However, the signal intensity and total area of fluorescence varied significantly between the treatment approaches. In the samples which underwent treatment with the Muc-2 antibody (without NP), a localized, discrete, and weak fluorescence signal appeared. On the contrary, a strong fluorescence signal was noticed in a large area upon treatment with NP-antibody. The results suggest that NPs grafted with FITC-labeled antibody are able to recognize tiny biochemical changes in the tissue through signal amplification.

The study has potential limitations that need to be addressed i) data is based on *ex vivo* experiments on biopsy specimens on a small number of BE patients, although it is expected that the experimental results will be very similar in the *in vivo* environment; ii) NPs without antibody were not used in the study; iii) the biopsy specimens were collected from confirmed BE patients with a higher degree of intestinal metaplasia, and therefore, the observed fluorescence may be higher than that observed in patients with early stage lesions; and iv) although *in vitro* analysis displayed no apparent cytotoxicity, safety concerns might come up in the event of *in-vivo* administration of NPs. However, the major strength of the current study is that for the first time the potential usage of NP for MEI and for diagnosis of specific gastrointestinal disease has been shown thereby opening the door for future applications in this field.

In conclusion, molecular endoscopic imaging using biodegradable, inorganic NPs has been proved to be an effective method for the detection of precancerous lesions. Thus, the proof of concept of our study is established. In the next phase, human trials should be conducted in order to validate the results clinically. In the final stage, the NPs can potentially also be loaded with cytotoxic materials for targeted therapy of lesions.

## Supporting information

S1 Fig CLE images before antibody/NPs application to SE and BE. (PDF)

S2 Fig CLE images of SE after antibody/NPs application. (PDF)

S3 Fig CLE images of BE after antibody/NPs application. (PDF)

## References

[1] Conteduca V, Sansonno D, Ingravallo G et al Barrett’s esophagus and esophageal cancer: An overview. *International Journal of Oncology* 2012; 41: 414–424. 10.3892/ijo.2012.1481 22615011

[2] Ahmed S, Strand S, Weinmann-Menke J et al Molecular endoscopic imaging in cancer. *Digestive Endoscopy* 2018 11; 30(6):719–29. 10.1111/den.13199 29846982

[3] Hossain MS, Chowdhury EH. Citrate- and Succinate-Modified Carbonate Apatite Nanoparticles with Loaded Doxorubicin Exhibit Potent Anticancer Activity against Breast Cancer Cells. *Pharmaceutics*. 2018 3; 11: 10 (1). 10.3390/pharmaceutics10010032 29534497 PMC5874845

[4] Vakil N, van Zanten SV, Kahrilas P et al The Montreal definition and classification of gastroesophageal reflux disease: a global evidence-based consensus. *Am J Gastroenterol*. 2006 8; 101(8):1900–20; quiz 1943. 16928254 10.1111/j.1572-0241.2006.00630.x

[5] Sturm MB, Joshi BP, Lu S et al Targeted imaging of esophageal neoplasia with a fluorescently labeled peptide: first-in-human results. *Sci Transl Med* 2013; 5:184ra61. 10.1126/scitranslmed.3004733 23658246 PMC3859345

[6] Atreya R, Neumann H, Neufert C et al In vivo imaging using fluorescent antibodies to tumor necrosis factor predicts therapeutic response in Crohn’s disease. *Nat Med*. 2014 3;20(3):313–8. 10.1038/nm.3462 24562382 PMC4479137

[7] Burggraaf J, Kamerling IMC, Gordon PB et al Detection of colorectal polyps in humans using an intravenously administered fluorescent peptide targeted against c-Met. *Nat Med* 2015;21:955–961. 10.1038/nm.3641 26168295

[8] Bird-Lieberman EL, Neves AA, Lao-Sirieix P et al Molecular imaging using fluorescent lectins permits rapid endoscopic identification of dysplasia in Barrett’s esophagus. *Nat Med*. 2012 1 15;18(2):315–21. 10.1038/nm.2616 22245781

[9] Huang HC, Barua S, Sharma G et al Inorganic nanoparticles for cancer imaging and therapy. *J*. *Control Release* 2011; 155: 344–357. 10.1016/j.jconrel.2011.06.004 21723891

[10] Mozar FS and Chowdhury EH. Surface-Modification of Carbonate Apatite Nanoparticles Enhances Delivery and Cytotoxicity of Gemcitabine and Anastrozole in Breast Cancer Cells. *Pharmaceutics* 2017 6; 9(2):21 10.3390/pharmaceutics9020021 28590445 PMC5489938

[11] Unterweger H, Janko C, Schwarz M et al Non-immunogenic dextran-coated superparamagnetic iron oxide nanoparticles: a biocompatible, size-tunable contrast agent for magnetic resonance imaging. *Int J Nanomedicine* 2017;12:5223–5238. 10.2147/IJN.S138108 28769560 PMC5533574

[12] Petros RA, DeSimone JM. Strategies in the design of nanoparticles for therapeutic applications. *Nat Rev Drug Discov* 2010; 9:615–27. 10.1038/nrd2591 20616808

