# Supplementary figures and images for: Molecular endoscopic imaging for the detection of Barrett’s metaplasia using biodegradable inorganic nanoparticles: An ex-vivo pilot study on human tissue
Source: PLoS One. 2020 Oct 1;15(10):e0239814. doi: 10.1371/journal.pone.0239814 (PMC7529275; doi:10.1371/journal.pone.0239814)

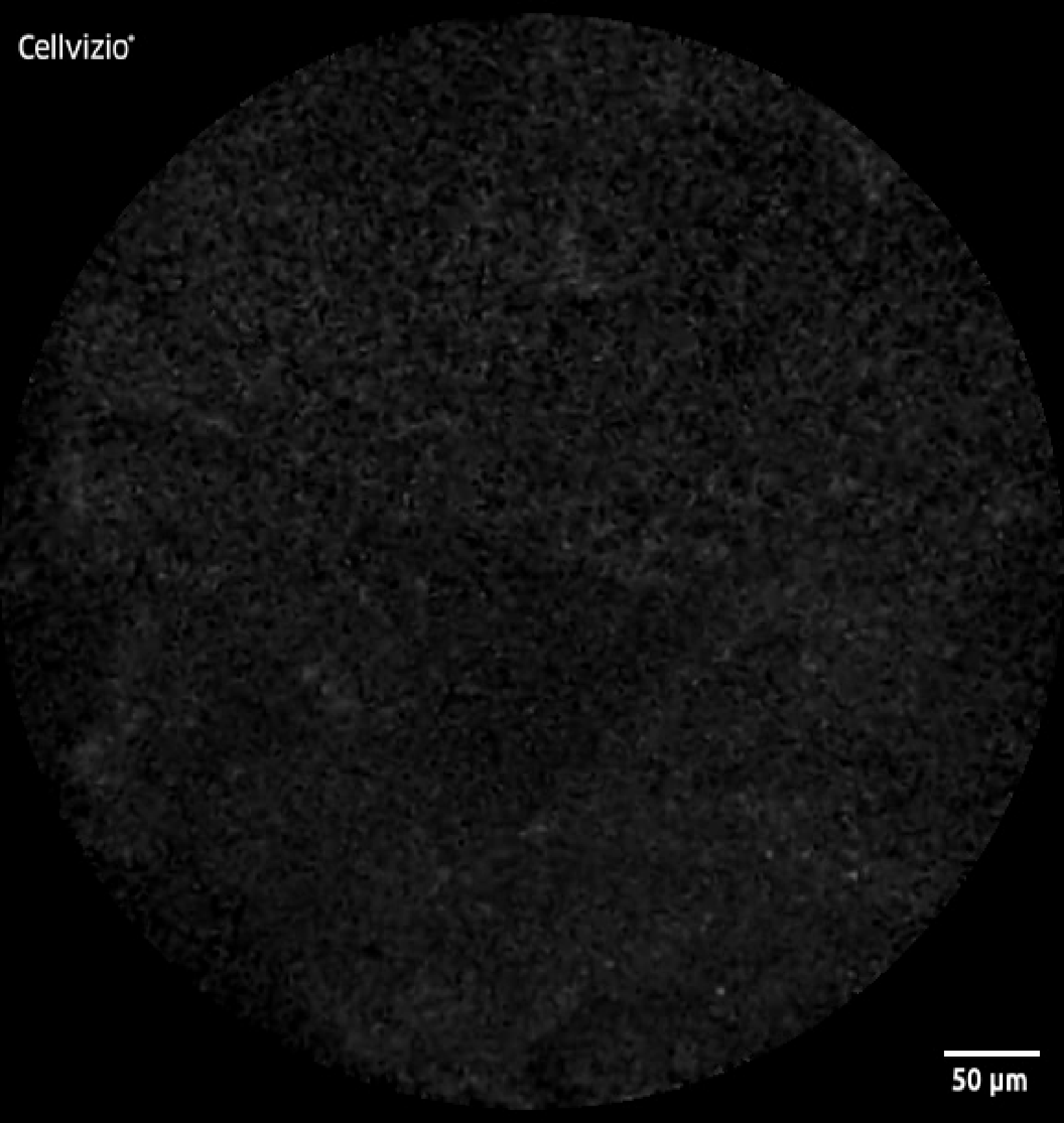

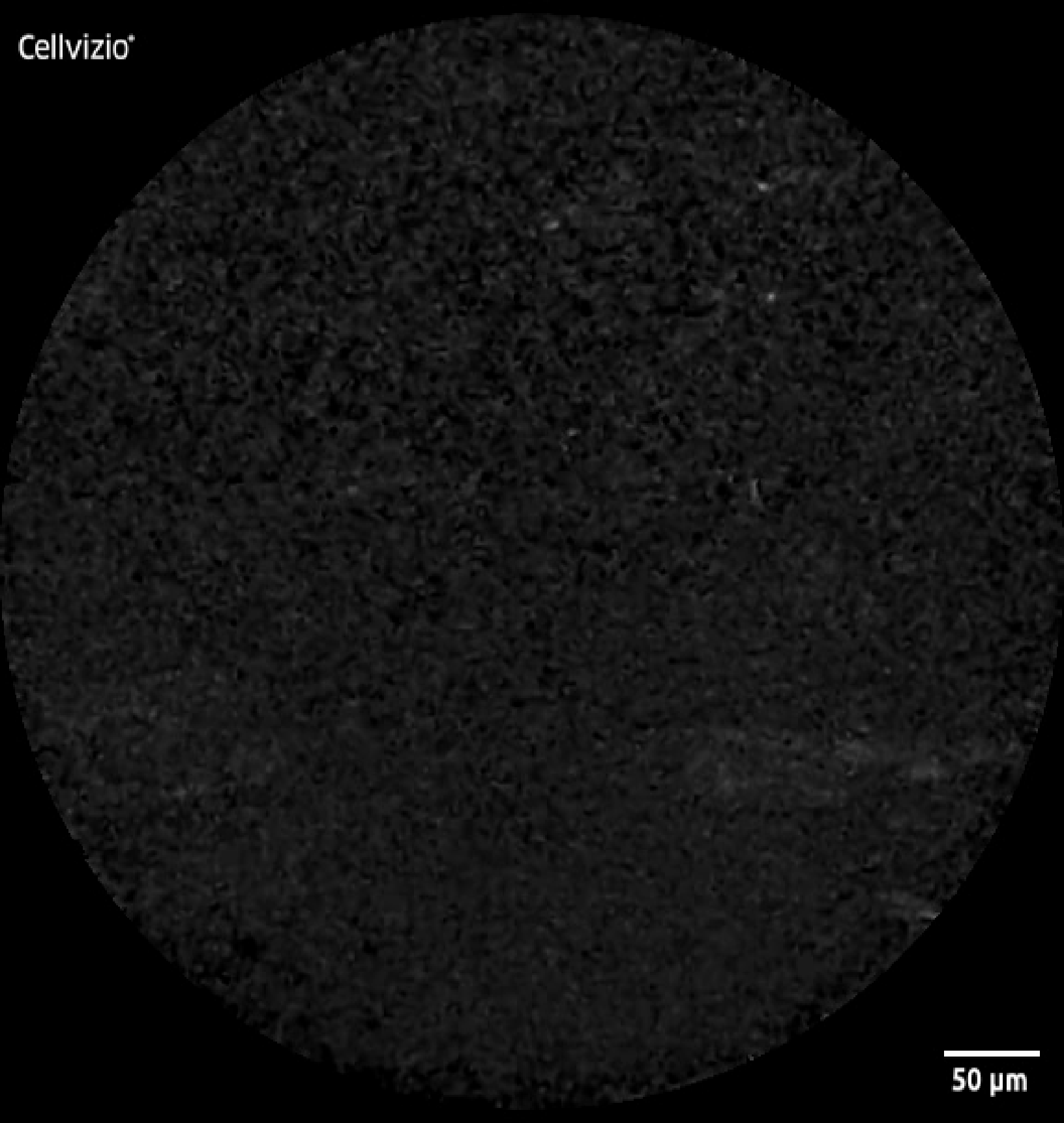

Supplement: S1 Fig — (PDF) [file pone.0239814.s001.pdf]

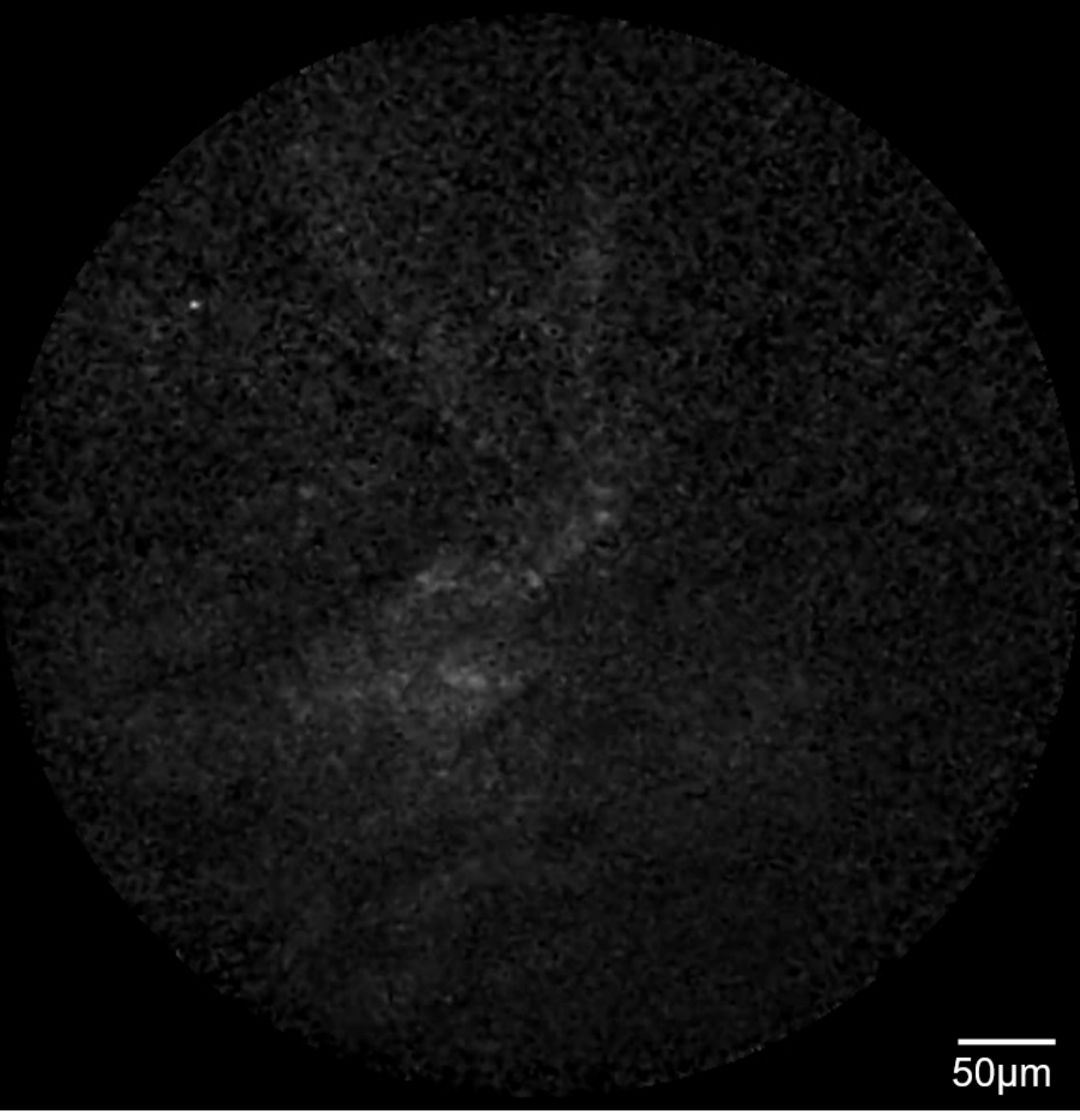

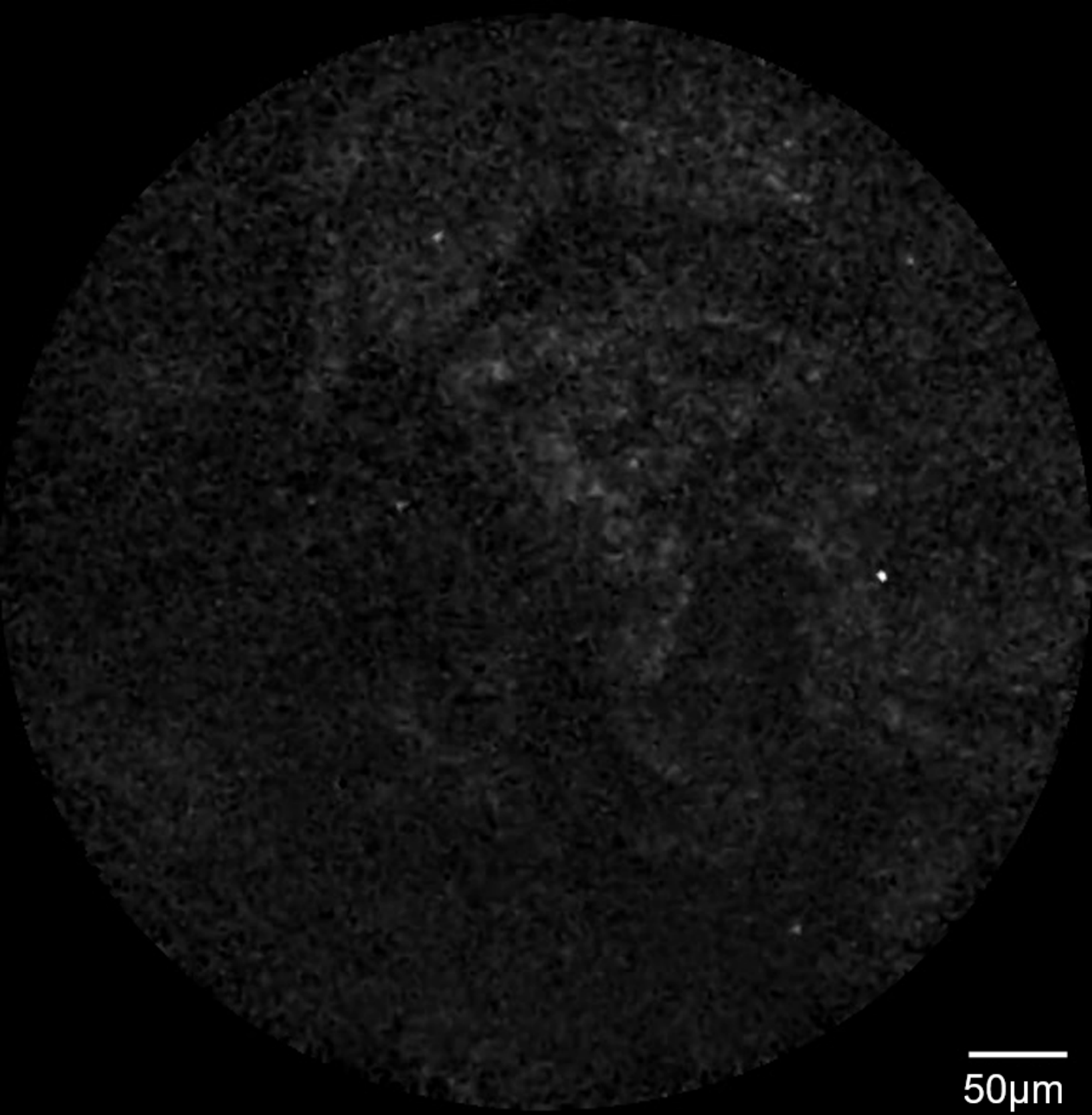

Supplement: S2 Fig — (PDF) [file pone.0239814.s002.pdf]

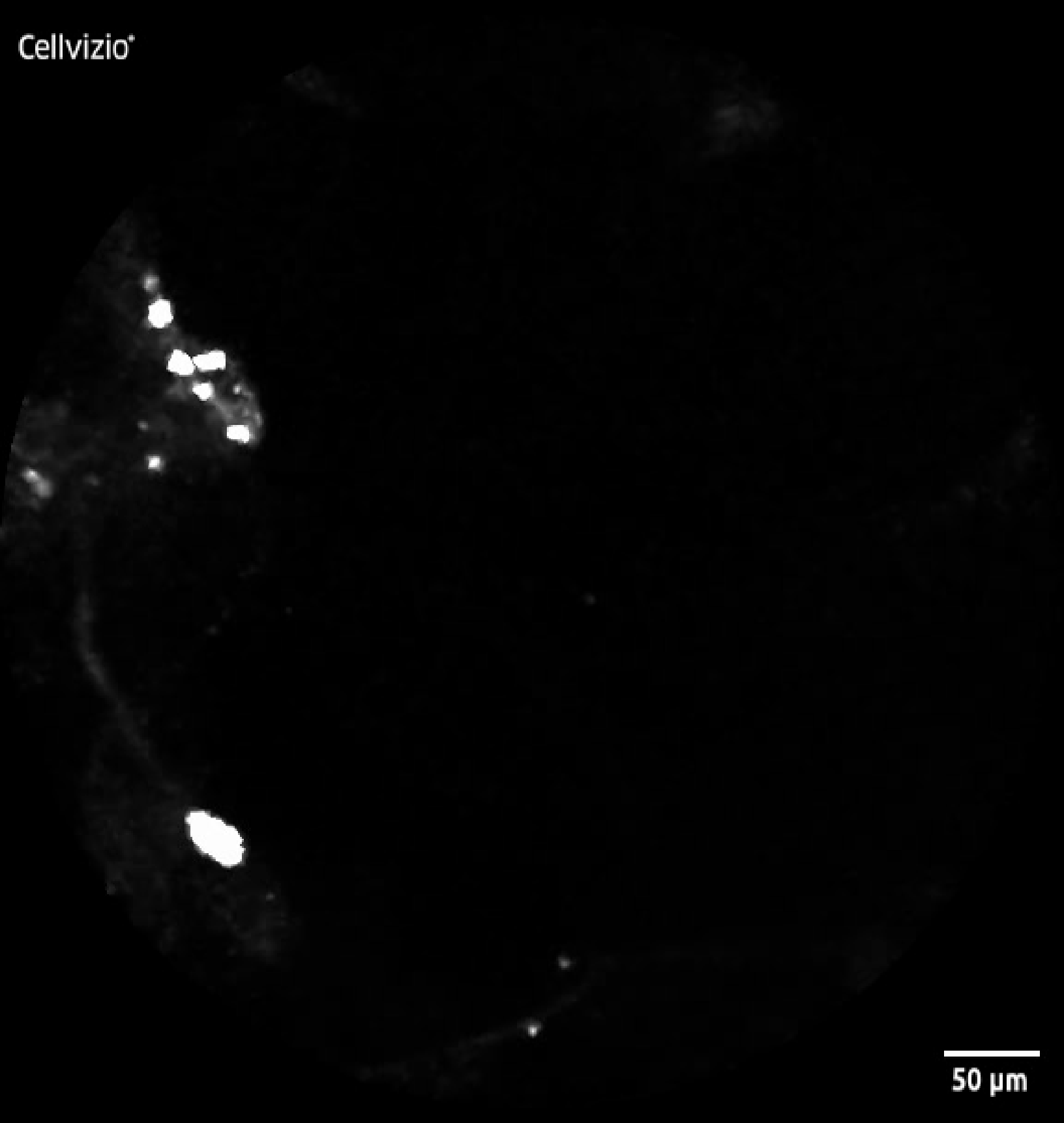

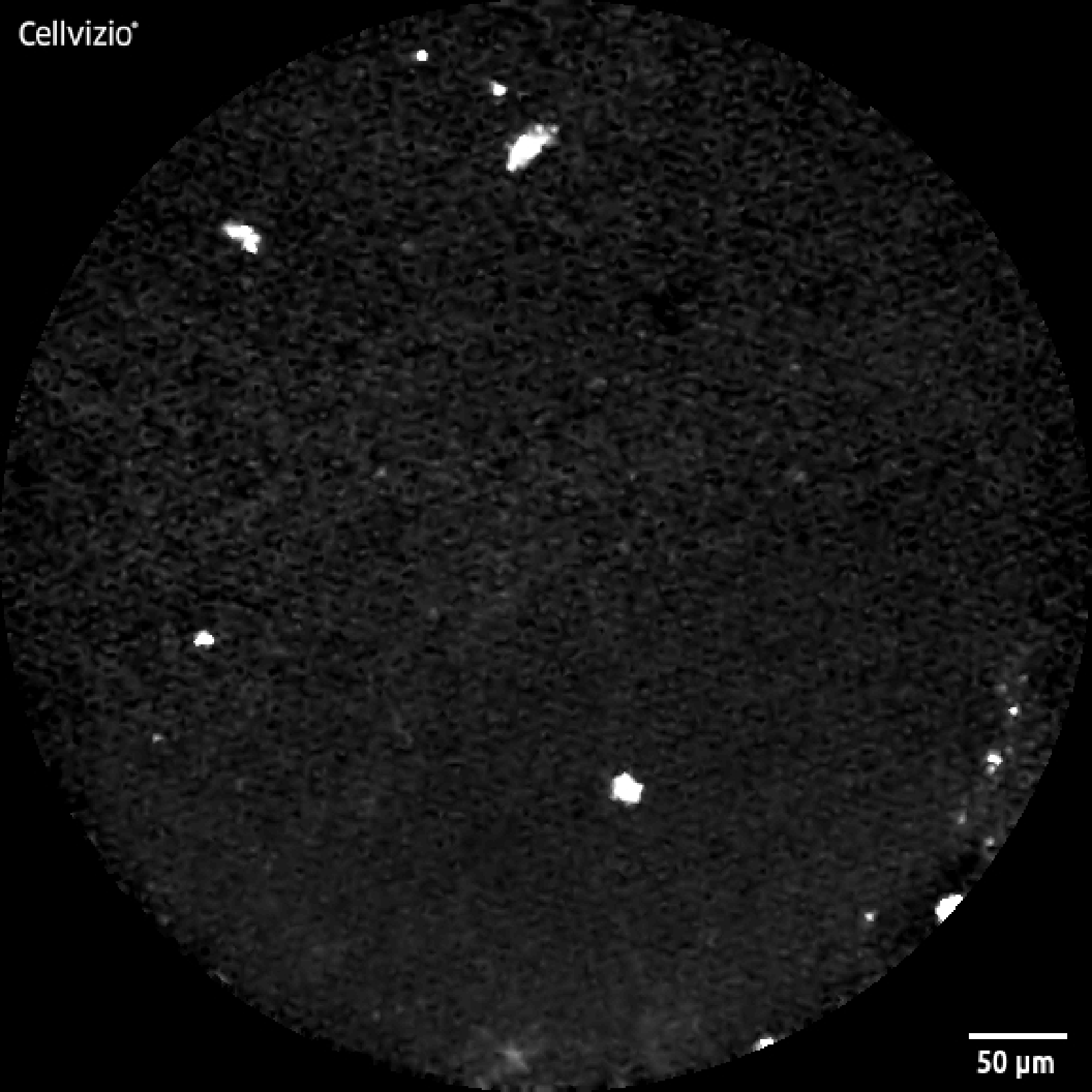

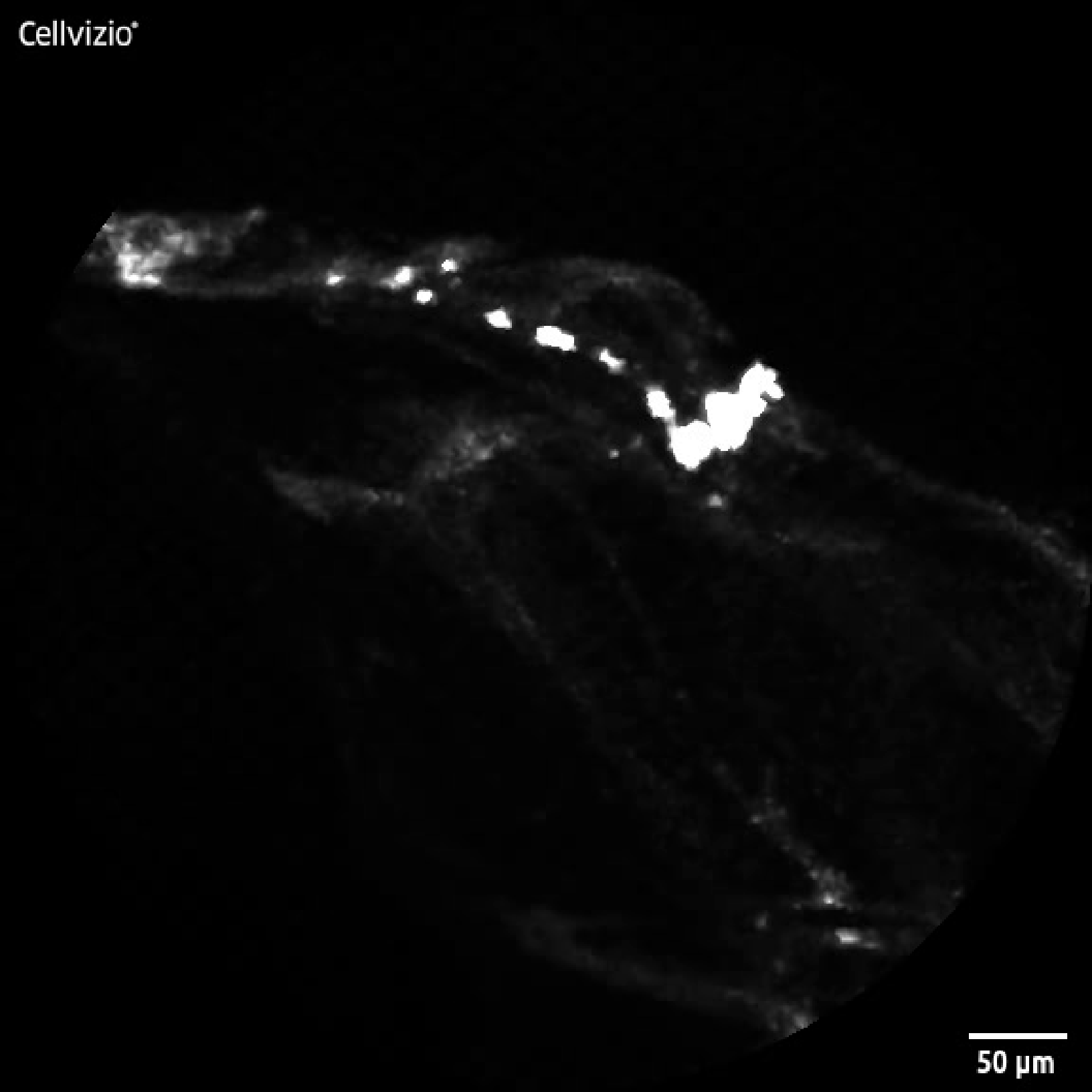

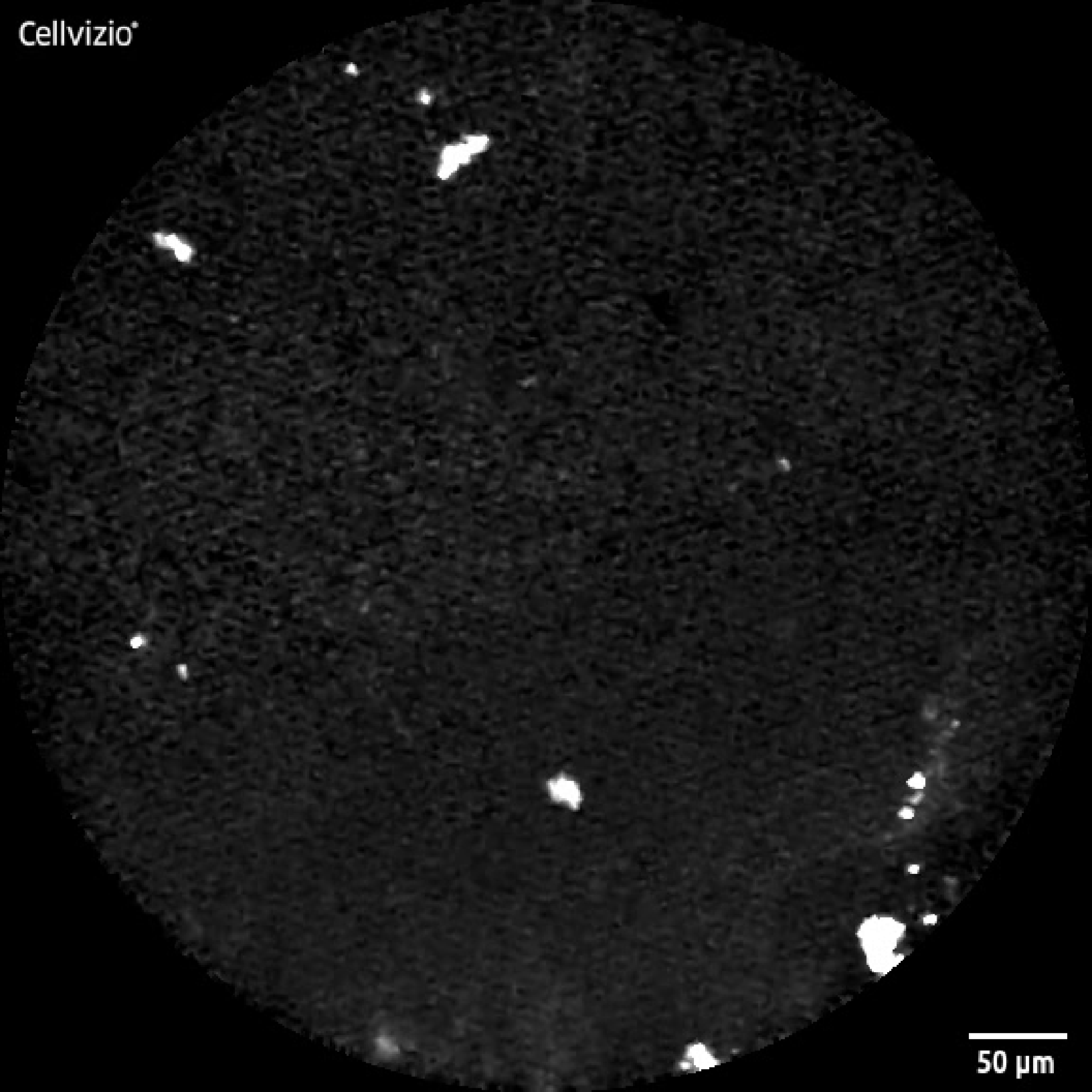

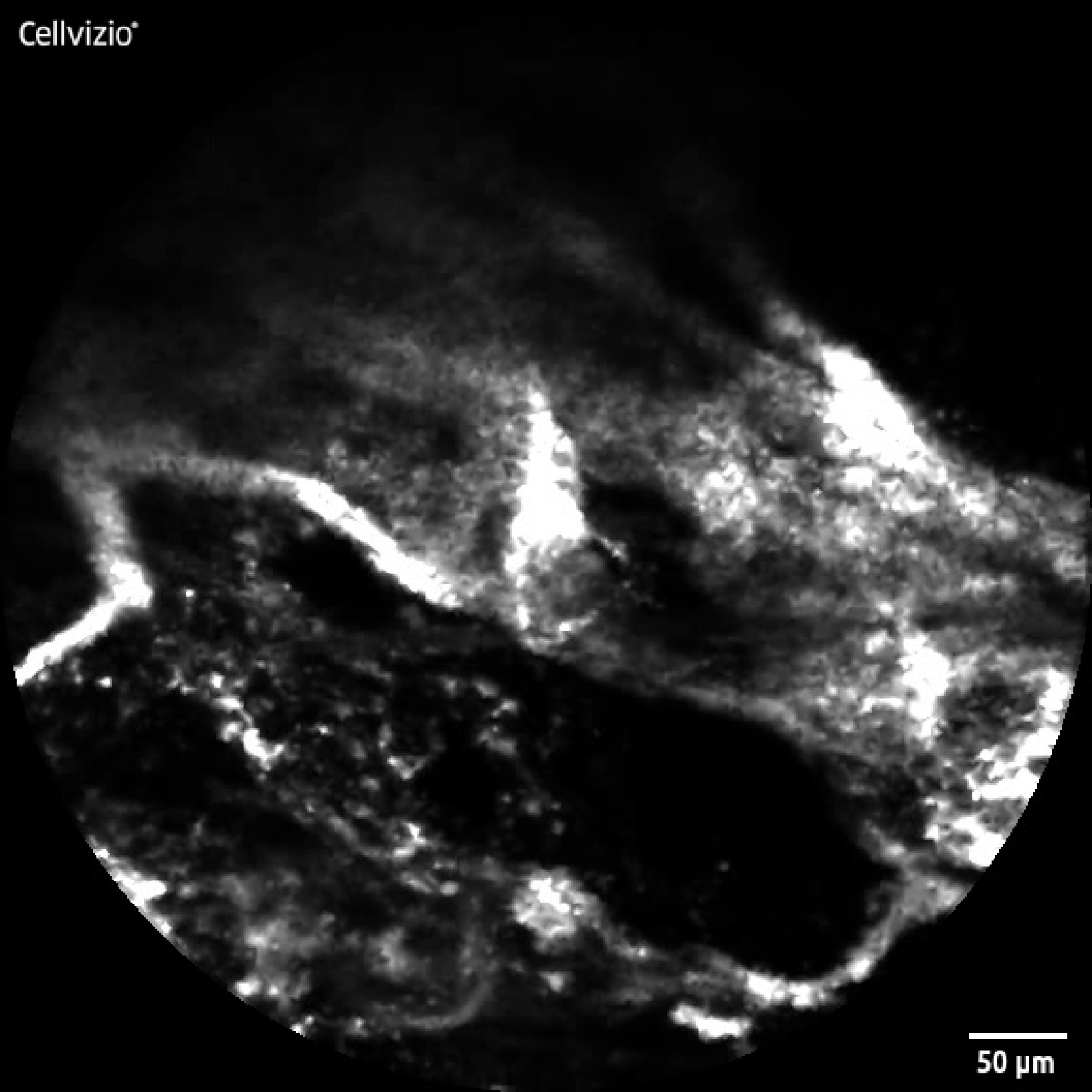

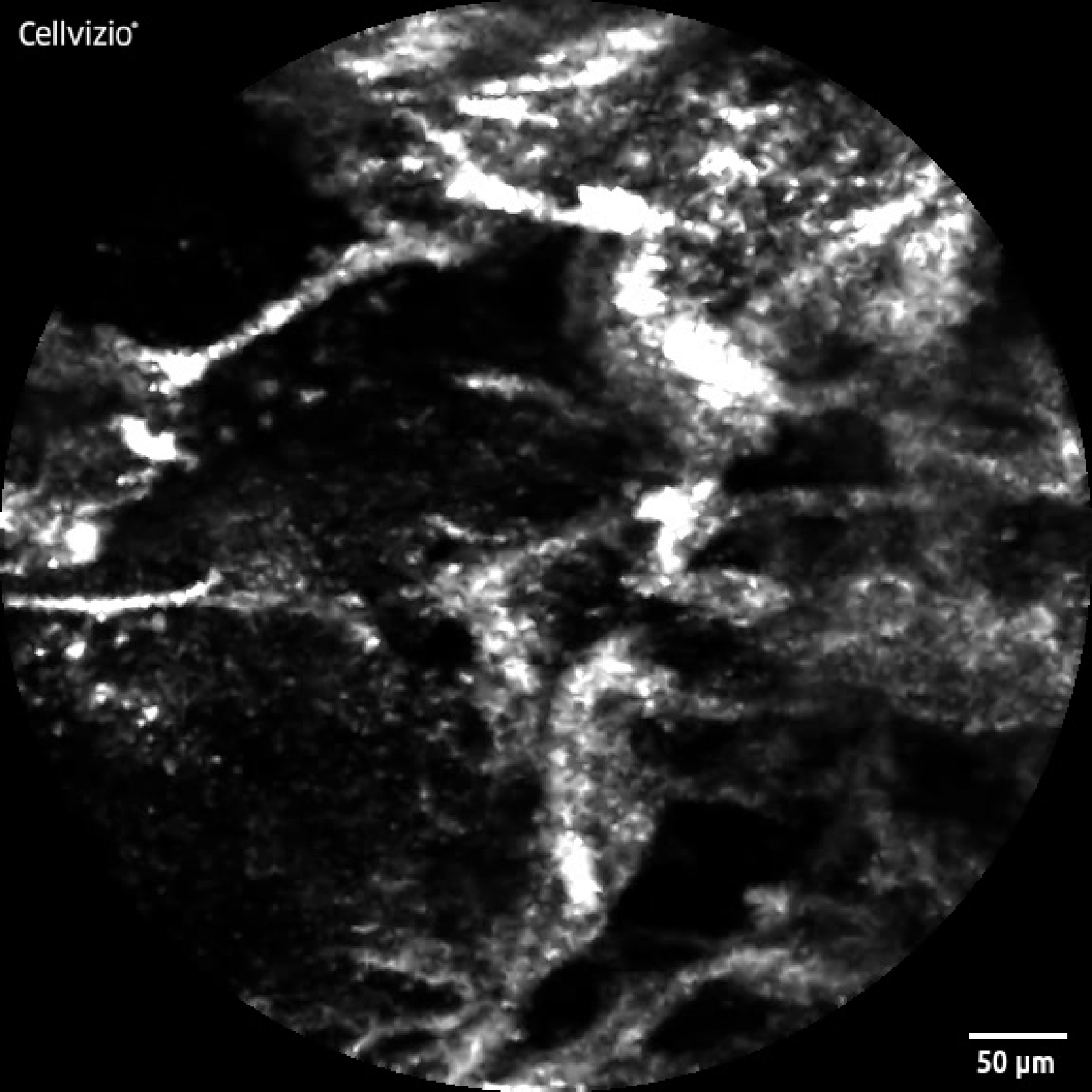

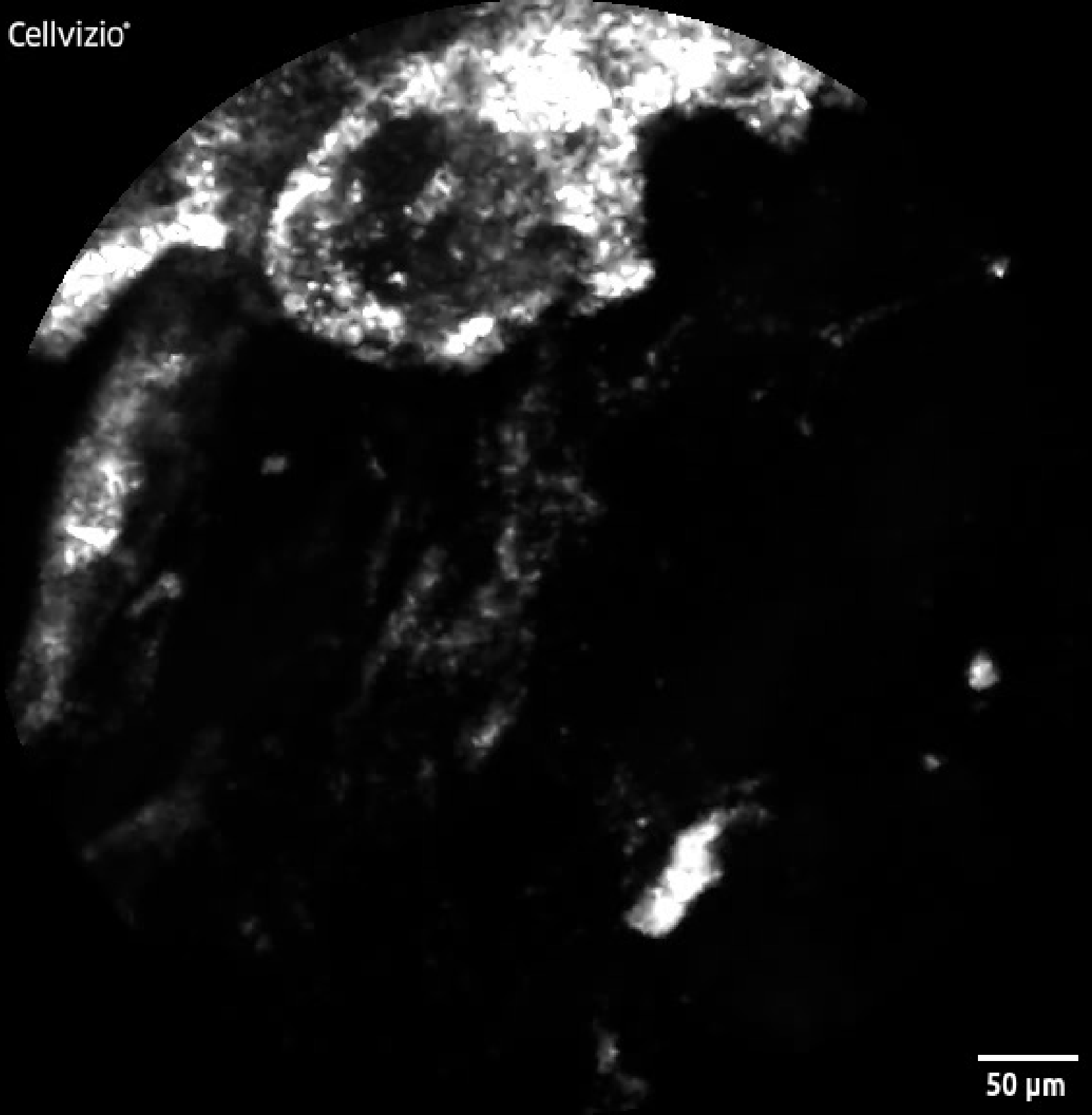

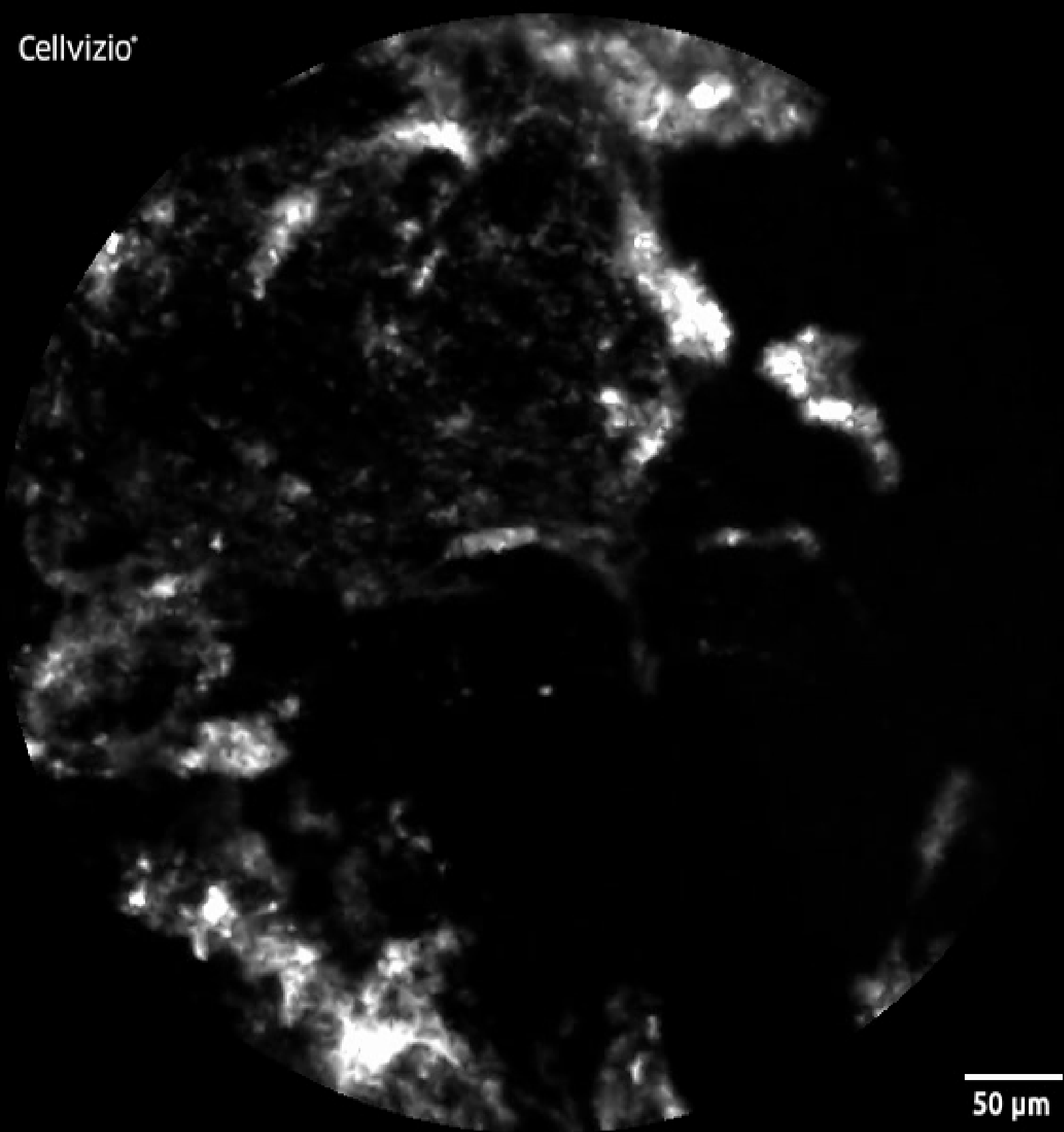

Supplement: S3 Fig — (PDF) [file pone.0239814.s003.pdf]
